# Elementary properties of Ca^2+^ channels and their influence on multivesicular release and phase-locking at auditory hair cell ribbon synapses

**DOI:** 10.3389/fncel.2015.00123

**Published:** 2015-04-08

**Authors:** Jacopo Magistretti, Paolo Spaiardi, Stuart L. Johnson, Sergio Masetto

**Affiliations:** ^1^Department of Biology and Biotechnology “Lazzaro Spallanzani”, University of PaviaPavia, Italy; ^2^Department of Brain and Behavioral Sciences, University of PaviaPavia, Italy; ^3^Department of Biomedical Science, University of SheffieldSheffield, UK

**Keywords:** inner hair cell, Ca^2+^ channel, patch-clamp, phase locking, cochlea, ribbon synapse, multivesicular release, Eps8

## Abstract

Voltage-gated calcium (Ca_v_1.3) channels in mammalian inner hair cells (IHCs) open in response to sound and the resulting Ca^2+^ entry triggers the release of the neurotransmitter glutamate onto afferent terminals. At low to mid sound frequencies cell depolarization follows the sound sinusoid and pulses of transmitter release from the hair cell generate excitatory postsynaptic currents (EPSCs) in the afferent fiber that translate into a phase-locked pattern of action potential activity. The present article summarizes our current understanding on the elementary properties of single IHC Ca^2+^ channels, and how these could have functional implications for certain, poorly understood, features of synaptic transmission at auditory hair cell ribbon synapses.

## Introduction

The mammalian auditory system has evolved specialized structures and cellular mechanisms that allow sound information to be relayed to the brain with unparalleled temporal precision. Our ability to localize sound sources in the environment depends on the preservation of timing accuracy along the auditory pathway. Low-frequency sounds are localized by specialized cells in the brainstem that compare the temporal delay between the phase-locked activity originating from the two ears, which can be as small as ten microseconds (Köppl, 1997; Carr and Macleod, 2010). The localization of high frequency sounds relies more on the detection of intensity differences between the outputs of the two ears, but the timing information obtained from the beginning and end of the sound envelope also has an important role (Moore, 1991; McAlpine, 2005). The intricate cellular mechanisms responsible for such exquisite temporal performance are still not well understood.

The initial step in the conversion of sound into an electrical signal takes place in the inner hair cells (IHCs), the primary sensory receptors of the cochlea, whereby sound-induced deflection of the hair bundle gates mechano-electrical transducer (MET) channels allowing the depolarizing flow of K^+^ ions into the cell. The interplay of the transducer current with the cells basolateral membrane currents generates the characteristic IHC receptor potential (Russell and Sellick, 1978), the size of which is graded to sound level. A fraction of MET channels are open at rest (Johnson et al., 2012), resulting in a tonic inflow of K^+^ which depolarizes the IHC *in vivo*. The *in vivo* resting membrane potential (*V*_m_) activates a proportion of voltage-gated Ca^2+^ channels that is thought to drive the tonic release of glutamate onto afferent terminals (Glowatzki and Fuchs, 2002). The resulting AMPA-receptor-mediated post-synaptic depolarization is converted into a resting discharge of action potentials in the afferent fiber that is referred to as its spontaneous rate (Liberman, 1978). Upon sound stimulation, a greater proportion of MET channels are opened, which increases IHC depolarization and consequently Ca^2+^ influx/transmitter release. Any given IHC contacts numerous afferent nerve terminals, which extend from cochlear ganglion cells. IHCs provide the sole synaptic input to those neurons. Moreover, each IHC contacts a given ganglion cell through a single synaptic site; thus, all the information the hair cell has to transmit to the neuron must be funneled through that one synaptic site (Trussell, 2002). IHCs’ synaptic sites contain a special structure called the “ribbon”, which is covered with synaptic vesicles (von Gersdorff, 2001). Ribbons are thought to ensure a continuous supply of vesicles to the plasma membrane for exocytosis during endless ongoing stimulation (Fuchs, 2005). Moreover, at the presynaptic side of each ribbon synapse a pool of 16–30 ready-to-release docked vesicles can be typically observed (Khimich et al., 2005), which might account for the multivesicular release events recorded postsynaptically (Glowatzki and Fuchs, 2002). One could expect that increasing IHC depolarization will result in an increase of the amplitude and frequency of these multivesicular events. Instead, large excitatory post-synaptic current (EPSCs) can be recorded at rest, the frequency, but not amplitude, of which increases with IHC depolarization (Glowatzki and Fuchs, 2002; Goutman and Glowatzki, 2007).

Also still largely unexplained is the transduction system’s ability to phase-lock the afferent spiking to a particular time point (phase) of the low-to-mid-frequency sinusoidal sound wave independent of its intensity (Rose et al., 1967; Fuchs, 2005; Goutman, 2012). Indeed, increasingly larger receptor potentials, driven by increasingly louder sounds, should cause more Ca^2+^ influx and increasingly faster vesicular fusion (Fuchs, 2005).

In this article we focus on the elementary properties of IHC Ca_V_1.3Ca^2+^ channels, which represent 90% of IHC Ca^2+^ channels (Platzer et al., 2000), and how they could underlie some of the still poorly understood characteristics of synaptic transmission at individual IHC ribbon synapses.

## Results and Discussion

### Ca^2+^ Channel Number and Open Probability

**Figure 1:**
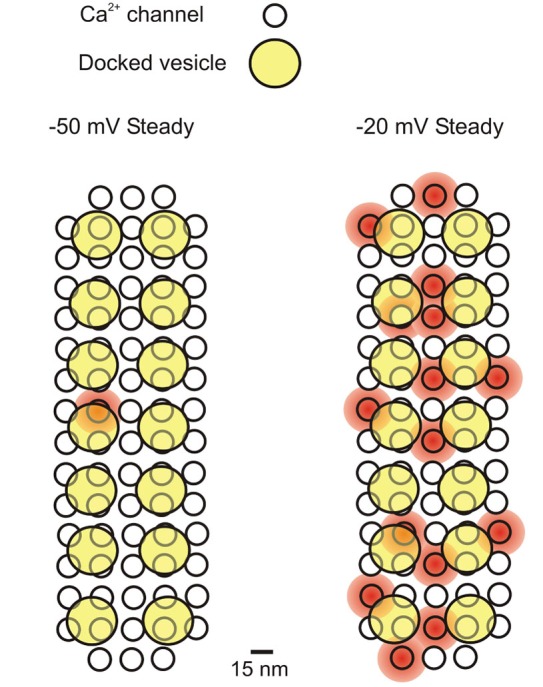
**Schematic representation of the Ca^2+^ influx-exocytosis coupling for a presynaptic active zone in a mature IHC**. Ca^2+^ channels simultaneously open at −50 mV and −20 mV (during a 500 ms depolarizing step from −70 mV), representing the average *P*_o_ found in cell-attached recordings: 0.01 and 0.21 at −50 and −20 mV, respectively (Zampini et al., 2013). It is assumed that each presynaptic site (active zone) contains 74 Ca^2+^ channels (see text) and 14 docked vesicles (Wong et al., 2014). The diameter of Ca^2+^ channels is ~15 nm (Wolf et al., 2003), while that of vesicles is ~40 nm (Khimich et al., 2005). Placing 74 Ca^2+^ channels closely packed (7.5 nm minimum distance) gives a presynaptic area of ~0.033 μm^2^, which is consistent with the most recent EM estimates (0.066 μm^2^; Khimich et al., 2005) and STED microscopy (0.11 μm^2^ and 0.097 μm^2^ for apical and middle cochlea IHCs, respectively: Meyer e al., 2009; 0.034 μm^2^ for mature IHCs: Wong et al., 2014). The shaded red circular areas indicate a 20 nanometer distance of cytosolic Ca^2+^ diffusion from the central pore of the Ca^2+^ channel when it is open. Kim et al. (2013) calculated that a Ca^2+^ channel current of 0.5 pA (we estimated 0.8 pA at −50 mV and 0.4 pA at −20 mV; Zampini et al., 2013) will produce a [Ca^2+^]_i_ of 350 μM near the channel mouth, which would decrease to 50–100 μM in a presynaptic region including three or four docked vesicles. Here we assumed that Ca^2+^ entering from each channel can reach at least one docked vesicle. If all Ca^2+^ channels showed an analogous gating behavior, then on average 1 (out of 74) Ca^2+^ channels (each with a *P*_o_ of 0.01) would be always open at −50 mV at the presynaptic site, and 15 (each with a *P*_o_ of 0.21) at –20 mV.

The total number of Ca^2+^ channels in a single IHC has been estimated in two ways: (1) using non-stationary fluctuation analysis of macroscopic (whole-cell) Ca^2+^ tail currents (Brandt et al., 2005; Vincent et al., 2014; Wong et al., 2014); and (2) by comparing the macroscopic (whole-cell) and elementary (cell-attached) Ca^2+^ current amplitudes (Zampini et al., 2010, 2013, 2014). The former uses the variability in size and shape of repeated deactivating Ca^2+^ tail currents (upon repolarization from a voltage step that activates the maximal macroscopic current) to estimate the channel number, single channel current and open probability (*P*_o_). The latter is a direct measure of elementary Ca^2+^ current (*i*_Ca_) size and *P*_o_ that is used to work out the number of channels from the size of the macroscopic Ca^2+^ current (*I*_Ca_). The number of Ca^2+^ channels per IHC estimated from fluctuation analysis is about 1, 800 (Brandt et al., 2005; Vincent et al., 2014; Wong et al., 2014). In cell-attached recordings, *P*_o_ was measured over long-lasting (500 ms) depolarizing voltage steps, in order to collect a large number of openings for reliable analysis. However, Ca^2+^ channels clearly underwent inactivation during these long pulses, as indicated by the shape of the ensemble-average currents (Zampini et al., 2013, 2014). If single channel analysis was limited to the initial 40 ms of the sweeps, *P*_o_ (at about –20 mV) increased from 0.024 to 0.06 in middle-turn IHCs (Zampini et al., 2014) and from 0.21 (Zampini et al., 2013) to 0.51 in basal-turn IHCs (data re-analyzed for the present study).^1^ The resulting total number of Ca^2+^ channels would be 6, 400 in adult middle turn IHCs and 1, 152 in adult basal turn IHCs. For basal-turn IHCs, from which we have collected most data, an average of 1, 152 Ca^2+^ channels per IHC would equate to 74 channels per synapse and 5 channels per vesicle (Figure 1), given that there are 14 ribbon synapses per cell (Zampini et al., 2013) and 10% of Ca^2+^ channels are believed to be extrasynaptic (Meyer et al., 2009). This is consistent with the model proposed by Wong et al. (2014) for apical-coil mouse IHCs where there are estimated to be up to 90 Ca^2+^ channels per release site.

### Ca^2+^ Channels and the Nanodomain

In a presynaptic nanodomain coupling of Ca^2+^ inflow—synaptic vesicle release (Stanley, 1993; Fedchyshyn and Wang, 2005), mathematical modeling predicts that a single Ca^2+^ channel may control vesicle release at each docking site (Weber et al., 2010). On the other hand, the opening of several Ca^2+^ channels may be necessary for the fusion of more distant vesicles at other synapses (microdomain; Borst and Sakmann, 1996). In IHCs, the linear Ca^2+^ dependence of exocytosis (Brandt et al., 2005; Johnson et al., 2005, 2009; Goutman and Glowatzki, 2007) has been explained using a nanodomain model for the coupling between Ca^2+^ channels and synaptic vesicles. In such a model, the Ca^2+^ sensor for vesicle fusion is located within a radius of tens of nanometers from the Ca^2+^ channel (Moser et al., 2006), as found in the squid giant synapse (Neher, 1998; Augustine, 2001; Oheim et al., 2006). The assumption is that Ca^2+^ influx through a single channel is sufficient to activate release of a nearby vesicle. With depolarization, the increase in Ca^2+^ channel *P*_o_ saturates the Ca^2+^ sensor, and therefore the postsynaptic response grows in linear proportion to the presynaptic Ca^2+^ current as additional Ca^2+^ channel openings bring with them their own “unit” of vesicular release. More direct evidence for the nanodomain coupling between Ca^2+^ channels and vesicle release sites in IHCs has come from the fact that the rapidly binding Ca^2+^ chelator BAPTA had a more prominent inhibitory effect on exocytosis than the more slowly acting EGTA (Moser and Beutner, 2000). The prediction is in fact that only the fast Ca^2+^ binding kinetics of BAPTA, but not the slower binding to EGTA, would be able to interrupt the action of Ca^2+^ in a nanodomain, as shown for the squid giant synapse (Adler et al., 1991; Augustine et al., 2003). However, Goutman and Glowatzki (2007) found that different concentrations of EGTA were able to slow the onset and rise time of the fast component of release at the hair cell afferent synapse, concluding that the Ca^2+^ sensor (likely to be on the vesicle) is approximately 23 nm from the Ca^2+^ channel. Because of the high affinity of the Ca^2+^ sensor (Beutner et al., 2001), one or few Ca^2+^ channel openings might still be sufficient for activating release, reconciling the effect of EGTA with a nanodomain model. Although the molecular identity of the Ca^2+^ sensor remains uncertain (Safieddine and Wenthold, 1999; Roux et al., 2006; Beurg et al., 2010; Johnson et al., 2010; Pangrsic et al., 2010), its binding to Ca^2+^ is highly cooperative, seemingly requiring the binding of five calcium ions to trigger release (Beutner et al., 2001). Vesicular release becomes more sensitive to Ca^2+^ inflow during IHC maturation, showing a more classical high-order dependence on Ca^2+^-current (*I*_Ca_) amplitude until the onset of hearing at around post-natal day 12, whereupon the release of synaptic vesicles becomes linearly dependent on *I*_Ca_ amplitude, as described above (Johnson et al., 2005, 2009). Some authors have hypothesized that linearization might depend on a change in the Ca^2+^-sensor properties leading to lowered Ca^2+^-binding cooperativity (Murphy et al., 2004; Thoreson et al., 2004; Johnson et al., 2005, 2010; Dulon et al., 2009). Heil and Neubauer (2010) have shown that, in principle, a linear dependence of synaptic release on Ca^2+^ influx, as that observed in adult hair cells, can emerge if different release sites, each one of which endowed with the same, supralinear Ca^2+^ sensitivity, are differently exposed to Ca^2+^ entering through voltage-gated Ca^2+^ channels. To fit real data, 75% to 90% of release sites were required to be exposed to 20- to 200-fold lower Ca^2+^ concentrations than those most effectively exposed to Ca^2+^ inflow. This would imply that in adult IHCs most releasable vesicles are under the control of Ca^2+^ microdomains (rather than nanodomains), or that an even looser spatial relation exists between them and voltage-gated Ca^2+^ channels. Until this is not demonstrated, such interpretation can be regarded as an interesting theoretical possibility. Morphologically, maturation-dependent linearization of vesicular release on *I*_Ca_ amplitude is accompanied by a change in the IHC active zone/postsynaptic density complexes that are initially multiple, small and spot-like and then become large single structures. There is also a topographical re-arrangement of Ca^2+^ channels, which matches that of the active zones: Ca^2+^ channels are initially located in several smaller, round clusters and then form a larger stripe-like cluster (Wong et al., 2014). Indeed, in the absence of Eps8, which plays a crucial role in the physiological maturation of mammalian cochlear IHCs, the developmental linearization of the exocytotic Ca^2+^ sensitivity in IHCs does not occur (Zampini et al., 2011).

The nanodomain interaction between the active Ca^2+^ channel and the Ca^2+^ sensor (Figure 1) seems necessary to ensure that Ca^2+^ provided by one or a few Ca^2+^ channels is sufficient to trigger vesicle release with minimal delay before it diffuses away or is buffered. However, the nanodomain scenario does not necessarily mean that *any* Ca^2+^-channel opening, however brief, will be sufficient to saturate the Ca^2+^ sensor and trigger vesicle release. The idea that the Ca^2+^ sensor is easily saturated by single Ca^2+^ channel openings implies that, given the channel’s mean open time (τ), the fraction of channel openings long enough to saturate the Ca^2+^ sensor will be dominant over the fraction of shorter, non-saturating, openings. For instance, if a channel open duration equals to one tenth of τ (i.e., 1.65 ms at –20 mV; Zampini et al., 2013) is sufficient to saturate the Ca^2+^ sensor, then ~90% of the openings will be long enough to bring about the same result. This would facilitate the phase-locking of vesicle release to sound frequencies of a few kHz (Palmer and Russell, 1986) where it would need to be triggered by depolarizing stimuli as short as the positive-going phase of the stimulus (a few hundreds microseconds) whilst maintaining a constant phase relationship (Pickles, 1996; Rossing, 2007). The sub-ms activation and deactivation kinetics of IHC Ca^2+^ channels (first channel openings are estimated to occur with a delay of about 50 μs in physiological conditions; Zampini et al., 2013, 2014), would be suitably rapid to encode sound onset and support phase-locking.

### Properties of EPSCs Generated by IHC Synapse Activation

Patch-clamp recordings from single nerve terminals contacting rat IHCs have shown that various types of EPSCs are evoked by IHC depolarization, from miniature EPSCs (mEPSCs) to large multi- or monophasic events seemingly resulting from the fusion of up to 20 vesicles (Glowatzki and Fuchs, 2002). The most abundant events (>70%), however, were large monophasic EPSCs (M-EPSCs), corresponding to the size of 3–6 summated mEPSCs (Glowatzki and Fuchs, 2002; Goutman and Glowatzki, 2007). M-EPSCs have also been observed in lower vertebrates, and presumed to result from the simultaneous release of multiple vesicles (Keen and Hudspeth, 2006; Suryanarayanan and Slaughter, 2006; Li et al., 2009; Schnee et al., 2013). Important for the following discussion, it has recently been suggested that M-EPSCs could underlie accurate phase-locking of spikes in the auditory fibers (Goutman, 2012; Li et al., 2014). In this section we attempt to reconcile the features of EPSCs with the properties of the single IHC Ca^2+^ channels.

The frequency of M-EPSCs during a step depolarization of IHCs increases in proportion to the stimulus amplitude, but at all voltages these large events appear to dominate over the lower-amplitude or less synchronous events. In immature rat IHCs, M-EPSC frequency was 2 Hz at –50 mV and 20 Hz at –20 mV (Goutman and Glowatzki, 2007) and seemed to be generally higher in more adult rats (Grant et al., 2010).

Under the assumption that mEPSCs are due to the release of single neurotransmitter quanta, and therefore correspond to the full fusion of single vesicles, the fact that M-EPSCs are the dominant postsynaptic events during sustained IHC depolarization suggests there is an intrinsic mechanism devoted to maximizing the release synchrony of a relatively homogeneous number of vesicles at IHC ribbon synapses. So far three models have been proposed for such a mechanism: (1) the ribbon itself, or proteins associated with the ribbon, facilitate the coordinated fusion of multiple vesicles docked at the active zone (Glowatzki and Fuchs, 2002; Singer et al., 2004); (2) the Ca^2+^ nanodomain around an open Ca^2+^ channel drives the simultaneous release of multiple vesicles (Jarsky et al., 2010; Graydon et al., 2011); and (3) multiple vesicles fuse together prior to fusing with the IHC membrane (termed compound fusion) (Matthews and Sterling, 2008). More recently, it has been proposed that the event corresponding to the full fusion of a single vesicle is in fact the M-EPSC, which would therefore represent a big-size elementary event, whereas the lower-amplitude EPSCs would result from incomplete or transitory fusion events, and multiphasic EPSCs from the flickering of the fusion pore between open and closed states (Chapochnikov et al., 2014). In the case of the incomplete or flickering fusion events, only small amounts of neurotransmitter would be released for every opening of the fusion pore, thus producing submaximal activation of postsynaptic receptors. Uniquantal release would also be consistent with the high rates of sound-driven spikes in spiral ganglion neurons (up to about Hz; Taberner and Liberman, 2005) given a maximal presynaptic release rate of about 700 Hz (Pangrsic et al., 2010)—see Figure 1 in Chapochnikov et al. (2014).

In a nanodomain scenario, hypotheses 2 and 3, as well as the model by Chapochnikov et al. (2014), would all be compatible with an M-EPSC being triggered by the opening of a single Ca^2+^ channel. In the case of hypothesis 1, the simultaneous opening of multiple Ca^2+^ channels, each associated with a different vesicle, could be required to activate the molecular machinery responsible for the coordinated, synchronous release of the same vesicle subset. It is unlikely, however, that multiple Ca^2+^ channel openings summate to produce M-EPSCs, given that M-EPSCs of similar amplitude are also observed, although at a much lower frequency, when the IHC is at rest, a membrane potential at which the probability of two or more Ca^2+^ channels being simultaneously open is negligible (Figure 1). Consistent with this hypothesis, EPSC size and shape heterogeneity persists when release probability is reduced by abolition of IHC Ca^2+^ influx (Chapochnikov et al., 2014), whereas synchronized M-EPSCs would be expected to become less represented in lower extracellular Ca^2+^.

### Burst Openings of Ca_V_1.3 Channels May Improve the Reliability of Synaptic Transmission in IHCs

Single-channel studies have revealed an interesting feature of Ca_V_1.3 channels. We found that these channels are generally reluctant to open, but when they do, they open in bursts and maintain a very high open probability for a substantial amount of time (Zampini et al., 2010, 2013, 2014). In our recordings, a given Ca^2+^ channel could remain closed for several consecutive 500 ms depolarizing sweeps, and then suddenly shift to a bursting mode in which prolonged sequences of openings interrupted by brief closings produced periods of activity with a *P*_o_ close to 1. Therefore, the majority of Ca^2+^ influx occurs via the bursting activity of Ca^2+^ channels, and is otherwise (i.e., outside bursts) negligible. Early studies on Ca_V_1.2 channels also showed different modes of gating (Hess et al., 1984; Nowycky et al., 1985) which were called mode 0 (closed), 1 (brief and rare openings) and 2 (unusually long openings), and it was suggested that the exit from mode 0 depended on the metabolic state of the cell (Nowycky et al., 1985; Kamp and Hell, 2000; Carabelli et al., 2001). Mode 2 is favored by BayK 8644 (Hess et al., 1984; Nowycky et al., 1985; Ceña et al., 1989). In our experiments BayK 8644 was normally added to the pipette solution to increase Ca^2+^-channel activity, but mode 2 was also observed in the absence of BayK 8644 (Zampini et al., 2010).

We also found that, with depolarization, single Ca^2+^ channel kinetics mainly differed for the increase in importance of the shortest mean closed time, while mean open and closed time constants were relatively unaffected by membrane voltage (Zampini et al., 2013). Therefore, it appears that depolarization mainly favors the passage of the channel from a reluctant-to-open to a willing-to-open state. Once it opens, the Ca^2+^ channel open probability is then dominated by the burst modality of gating. This gating behavior suggests that at a given membrane potential the majority of Ca^2+^ channels are almost inactive (in modes 0 and 1), with the few channels opening in bursts (mode 2) underlying the majority of the macroscopic current recorded in whole-cell.^2^ Depolarization increases the chance that additional Ca^2+^ channels enter mode 2, accounting for the increase in the macroscopic current.

The likelihood that the majority of Ca^2+^ entry occurs during the burst opening of Ca^2+^ channels would have important implications as far as signal transmission at IHC afferent synapses is concerned. As discussed above, various lines of evidence suggest that IHC Ca^2+^ channels and release sites are within the spatial range of nanodomains, such that the opening of one channel might be sufficient to saturate the Ca^2+^ sensor and trigger vesicle release. One could hypothesize that, under these conditions, the most effective modality for Ca^2+^ channel opening in terms of vesicle release is burst opening. However, it is unlikely that the prolonged entry of Ca^2+^ during a burst is the necessary factor for vesicle release, since depolarizations of only a few hundred microseconds must be sufficient to trigger a release event during mid-frequency sound stimulation (see above).

**Figure 2:**
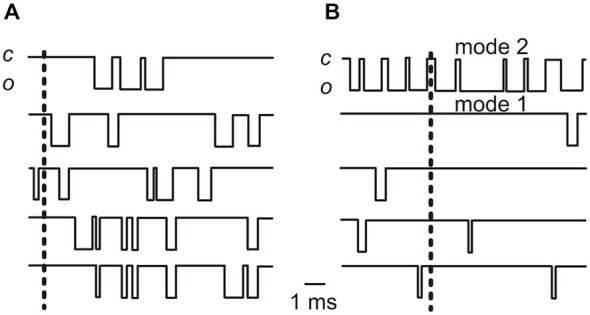
**Idealized traces showing elementary Ca^2+^ channel currents. (A)** The traces show the activity of five Ca^2+^ channels each opening with a *P*_o_ of 0.2 (a value similar to the average *P*_o_ found in cell-attached recordings at −20 mV in adult IHCs: Zampini et al., 2013). **(B)** The activity of five Ca^2+^ channels with one opening with a *P*_o_ of 0.8 (gating mode 2; top trace) and 4 channels opening with a *P*_o_ of 0.05. Note that in this case the average *P*_o_ for the 5 channel group is 0.2, as it is in **(A)**. In both **(A)** and **(B)** the vertical dashed lines indicate time points at which no Ca^2+^ channels are open (see text). Note that the simultaneous occurrence of no openings in all 5 channels is clearly more frequent in **(A)** than in **(B)**. By convention, the inward current is indicated by the downward deflection of the trace; “*c*” is closed, “*o*” is open.

It is more likely that a predominance of burst openings would improve the reliability of vesicle release. Let us assume, for example, that five Ca^2+^ channels are associated with a release-ready vesicle (see calculations above), with and average *P*_o_ across all 5 channel of 0.2 (Figure 2A). This average *P*_o_ could result from every one of the 5 channels having a *P*_o_ of 0.2; or it could result from 4 out of 5 channels opening with a *P*_o_ of 0.05, and one channel having a *P*_o_ of 0.8 (corresponding to a burst) (Figure 2B). In the former scenario, the probability of no channels opening would be (1–0.2)^5^ = 0.33, whereas in the latter it would *be* (1–0.05)^4^·(1–0.8) = 0.16. In this example, a single bursting channel, even when the group of channels shows the same average *P*_o_ as the non-bursting channels, would increase the probability of at least one Ca^2+^ channel being open at each active zone, from 0.67 to 0.84, which would increase the overall reliability of vesicle release.

### Ca^2+^ Channel Properties and Phase-Locked Exocytosis

**Figure 3:**
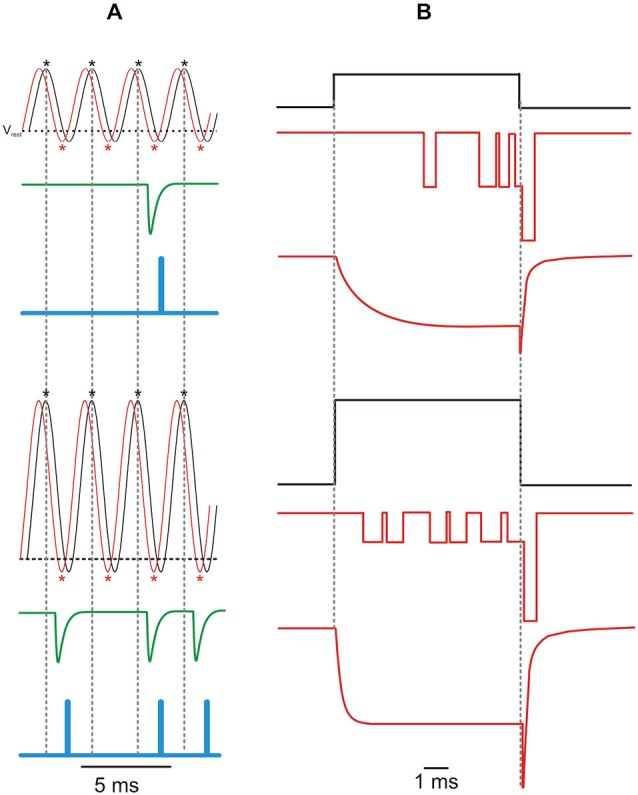
**Phase-locking and the Ca^2+^ current. (A)** Idealized traces showing a sinusoidal receptor potential (V_MET_; black line) and resulting Ca^2+^ current (I_Ca_; red line) in response to a 400 Hz sound wave with a low (upper panel) and high (lower panel) intensity. The horizontal dashed line indicates the adult mouse IHC resting membrane potential (V_rest_ = −60 mV; Johnson et al., 2011). Asterisks indicate peak of I_Ca_ (red) and of V_MET_ depolarization (black). The resulting increase in intracellular [Ca^2+^] elicits phase-locked M-EPSCs (green trace; M-EPSC re-drawn from Glowatzki and Fuchs, 2002) which are encoded in action potentials at the primary auditory afferents (blue trace). One EPSC can trigger only one spike (Rutherford et al., 2012), while no vesicles would be released during interspike intervals (i.e., spike discharge would reflects vesicle release probability; Moezzi et al., 2014). As a result of the increased Ca^2+^ channel *P*_o_ with IHC depolarization, louder sounds (bottom panel) elicit more action potentials (less failures) with the same timing (phase-locking), as indicated by the vertical dashed lines. Note that, as shown in the bullfrog auditory papilla (Figure 5 in Li et al., 2014), I_Ca_ phase-lags V_MET_, while M-EPSCs only occur in the V_MET_ repolarizing phase. Action potentials further lag V_MET_ due to the time required by electrotonic currents to depolarize the encoder region up to the spiking voltage threshold (~0. 5 ms; Rutherford et al., 2012). **(B)** Idealized elementary and macroscopic Ca^2+^ currents elicited by a voltage step from the resting membrane potential of −70 mV to −50 mV (top) or −20 mV (bottom). Activation kinetics of the macroscopic current re-drawn from Johnson and Marcotti (2008), deactivation kinetics re-drawn from Zampini et al. (2014). Increasing IHC depolarization reduces the latency and the time-to-peak of i_Ca_ and I_Ca_, while it decreases or increases the amplitude of i_Ca_ or I_Ca_, respectively.

Afferent fibers innervating apical- and middle-turn cochlear IHCs show phase-locked spiking activity to sound frequencies up to a few kHz, independent of stimulus intensity (Figure 3A; Rose et al., 1967). The independence of phase-locking on intensity is hard to reconcile with either the Ca^2+^ microdomain or nanodomain control of IHC exocytosis. In a microdomain, Ca^2+^ influx through several Ca^2+^ channels at a presynaptic site would resemble the macroscopic *I*_Ca_. Since *I*_Ca_ amplitude and activation speed increase with depolarization (Figure 3B), it is expected that neurotransmitter release will occur sooner the more intense the stimulus. In other words, increasing stimuli should produce progressively more phase-advanced afferent responses. A decrease in synaptic delay with increasing depolarization has been seen in rat IHCs (Goutman, 2012; see also Li et al., 2014 for frog auditory papilla hair cells). In a nanodomain, on the other hand, the latency-to-first Ca^2+^ channel opening decreases significantly with depolarization (Figure 3B). Therefore, either in a micro- or a nanodomain, depolarization would shorten the delay for Ca^2+^-dependent exocytosis and modify the phase of the response, interfering with phase-locking to that particular sound frequency.

A mechanism by which the accuracy of afferent phase-locking could be preserved independent of sound intensity has been proposed, involving the balance between short-term facilitation and depression of transmitter release at the hair cell ribbon synapse (Cho et al., 2011; Goutman, 2012). In this hypothesis, Ca^2+^ channel facilitation that occurs at voltages close to the resting membrane potential is compensated by synaptic vesicle depletion during prolonged trains of activity, producing a constant synaptic delay despite varying stimulus intensity (Goutman, 2012). However, a phase-advance should still be observed for the first cycles of the response before vesicle depletion occurs, whereas the first latencies (latency of the responses to first cycles) showed the same phase for all events (Goutman, 2012). This model, moreover, would imply that the afferent response to the first cycle/s of the sound wave would occur at different times depending on the sound level.

**Figure 4:**
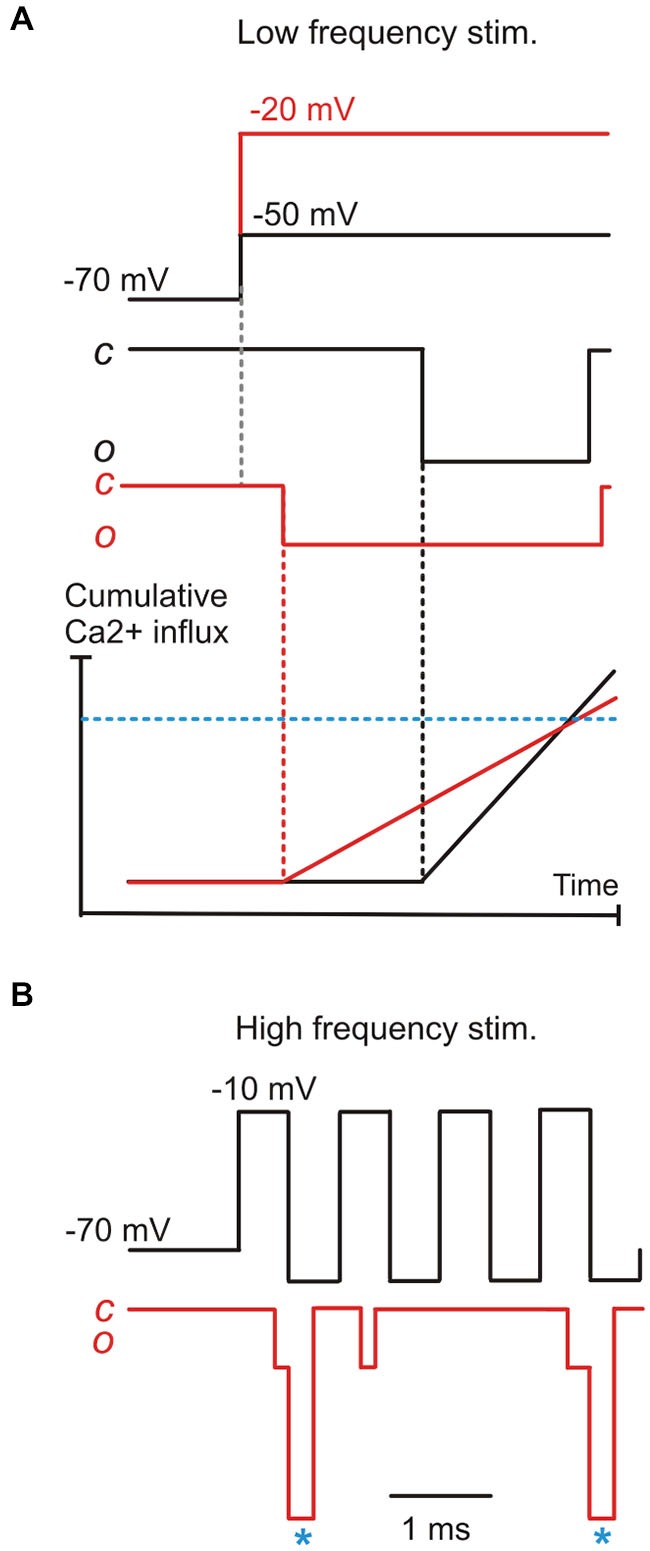
**A model for phase-locking based on the properties of elementary Ca^2+^ currents. (A)** the combined effects of the decrease in first latency and the decrease of elementary Ca^2+^-current amplitude brought about by IHC depolarization, resulting in a hypothetical “threshold” Ca^2+^ concentration for exocytotic Ca^2+^-sensor saturation (blue horizontal dashed line) being reached with similar timing as for a lower depolarization. This model would only work if vesicle release is governed by a single Ca^2+^ channel coupled in a nanodomain to the vesicle/Ca^2+^ sensor. **(B)** During high-frequency stimuli (e.g., 1 KHz), signal transmission likely depends upon elementary tail current (asterisks), particularly for high-intensity stimuli where the elementary current would be minimized by the smaller I_Ca_ driving force.

The properties of single Ca^2+^ channel currents could underlie the preservation of phase-locked transmission in a nanodomain in two possible ways. In the first scenario, as the IHC depolarizes, the amplitude of *i*_Ca_ decreases due to the reduction in driving force for Ca^2+^ entry (Figure 3B; Zampini et al., 2013). This would counteract the more rapid channel opening, such that the delay to Ca^2+^ sensor saturation for exocytosis may be comparable at different stimulus levels. This mechanism would allow the preservation of a constant phase relationship to changing sound intensity (Figure 4A). In a second scenario, a constant phase relation could be maintained by the effect of IHC repolarization on *i*_Ca_. During the repolarizing phase of a rapid cyclic stimulus, the large increase in driving force for Ca^2+^ would increase the amplitude of the current flowing through an already open Ca^2+^ channel. If repolarization is sufficiently fast, the amplitude of the elementary Ca^2+^ current would quickly rise to levels high enough to promptly saturate the Ca^2+^ sensor. These “saturation events” will cluster in a limited time window during the repolarizing phase, regardless of the amplitude of the foregoing depolarization, as long as it has opened the Ca^2+^ channel. During the macroscopic Ca^2+^ “tail” currents elicited by fast repolarization, the amplitude of elementary currents would be maximized with minimal jitter (Figure 3B). Their duration would also be short, since channel deactivation is faster than activation, which would be favorable for phase-locking towards the upper sound frequency limit (Figure 4B). Recent studies have shown that when IHCs are stimulated with a voltage sinusoid to mimic sound, the largest M-EPSCs occurred most frequently during the repolarizing rather than the depolarizing phase (see e.g., Figure 3 in Goutman, 2012; Figure 5 in Li et al., 2014), even when the synaptic delay (0.7–0.8 ms; Palmer and Russell, 1986) is taken into account. During high-frequency stimulation, the maximal speed of macroscopic Ca^2+^ current development occurs during the early repolarizing phase of the cycle (Figure 5 in Li et al., 2014). Tail Ca^2+^ currents have been shown to elicit time-locked M-EPSCs (Goutman, 2012). Finally, M-EPSCs elicited by depolarizing voltage steps delivered to rat IHCs (Goutman, 2012) and frog auditory hair cells (Graydon et al., 2014) appear better phase-locked during the Ca^2+^ tail current upon repolarization rather than to the peak *I*_Ca_. In the amphibian papilla, in which each afferent fiber receives input from several synaptic ribbons, the faster, less variable tail Ca^2+^ currents would seem better suited for triggering synchronous vesicle fusion than the stochastic opening of Ca^2+^ channels at different presynaptic sites. Therefore, in this second scenario, although IHC depolarization is necessary to open Ca^2+^ channels, subsequent IHC repolarization appears optimally suited for synchronizing post-synaptic activity with sound frequencies around the phase-locking limit.

In conclusion, it is possible that the elementary properties of IHC Ca^2+^ channels underlie several as yet unexplained features of the afferent response. The sub-ms first latency would ensure that at least some Ca^2+^ channels open with very short delay, allowing even relatively high frequency stimuli to be followed. The bursting behavior of Ca^2+^ channels, on the other hand, would increase the reliability of signal transmission. Finally, in a nanodomain where vesicle fusion is controlled by a single Ca^2+^ channel, the elementary current amplitude and opening latency could balance each other to produce constant phase-locking of the afferent response despite variations in sound intensity. The elementary tail currents would provide a rapid and large Ca^2+^ influx at the highest sound frequencies possible.

## Author and Contributors

JM, PS, SLJ, and SM substantially contributed to the conception or design of the work, analysis and/or interpretation of data, drafting of the text and /or figures, revision, final approval of the version to be published and agree to be accountable for all aspects of the work in ensuring that questions related to the accuracy or integrity of any part of the work are appropriately investigated and resolved.

## Conflict of Interest Statement

The authors declare that the research was conducted in the absence of any commercial or financial relationships that could be construed as a potential conflict of interest.

## Footnotes

^1^These values have been obtained in the presence of 5-μM BayK 8644, which stabilizes Ca_V_1-channel openings and increases their *P*_o_. In the absence of BayK 8644, *P*_o_ was approximately two-fold lower. Since, however, both whole-cell and cell-attached recordings were obtained in the presence of BayK 8644, the estimated number of Ca^2+^ channel would not be invalidated. BayK 8644 is also routinely used in fluctuation-analysis studies to increase *P*_o_, which is favorable for estimating the channel number. Under such conditions, a maximal *P*_o_ of ~0.8 has been estimated (Brandt et al., 2005; Vincent et al., 2014; Wong et al., 2014).

^2^Assuming that the same channel activity patterns also occur during whole-cell recording, the proportion of channels opening according to the high-*P*_o_, burst mode would conceivably dominate the macroscopic Ca^2+^ currents, including tail currents. Therefore, only the subset of channels operating in the high-*P*_o_ mode would be considered when fluctuation analysis is carried out.
